# Reining in BTK: Interdomain Interactions and Their Importance in the Regulatory Control of BTK

**DOI:** 10.3389/fcell.2021.655489

**Published:** 2021-06-23

**Authors:** Lauren E. Kueffer, Raji E. Joseph, Amy H. Andreotti

**Affiliations:** Roy J. Carver Department of Biochemistry, Biophysics and Molecular Biology, Iowa State University, Ames, IA, United States

**Keywords:** Bruton’s tyrosine kinase, B cell lymphoma, tyrosine kinase regulation, drug resistance, drug targeting approaches, BTK autoinhibition, Ibrutinib, BTK inhibitors

## Abstract

Since Dr. Ogden Bruton’s 1952 paper describing the first human primary immunodeficiency disease, the peripheral membrane binding signaling protein, aptly named Bruton’s tyrosine kinase (BTK), has been the target of intense study. Dr. Bruton’s description of agammaglobulinemia set the stage for ultimately understanding key signaling steps emanating from the B cell receptor. BTK is a multidomain tyrosine kinase and in the decades since Dr. Bruton’s discovery it has become clear that genetic defects in the regulatory domains or the catalytic domain can lead to immunodeficiency. This finding underscores the intricate regulatory mechanisms within the BTK protein that maintain appropriate levels of signaling both in the resting B cell and during an immune challenge. In recent decades, BTK has become a target for clinical intervention in treating B cell malignancies. The survival reliance of B cell malignancies on B cell receptor signaling has allowed small molecules that target BTK to become essential tools in treating patients with hematological malignancies. The first-in-class Ibrutinib and more selective second-generation inhibitors all target the active site of the multidomain BTK protein. Therapeutic interventions targeting BTK have been successful but are plagued by resistance mutations that render drug treatment ineffective for some patients. This review will examine the molecular mechanisms that drive drug resistance, the long-range conformational effects of active site inhibitors on the BTK regulatory apparatus, and emerging opportunities to allosterically target the BTK kinase to improve therapeutic interventions using combination therapies.

## Introduction

**FIGURE 1:**
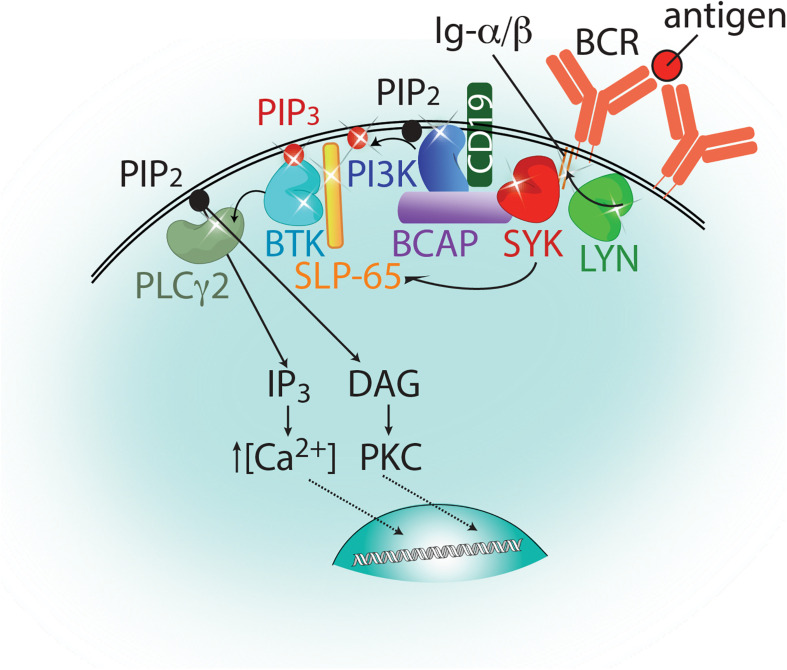
Proximal signaling events emanating from the B cell receptor. Antigen binding to the B cell receptor (BCR, orange) leads to the activation of a trio of kinases: LYN, SYK, and BTK, which are represented as bi-lobed structures colored lime green, red, and cyan, respectively. Phosphorylation events carried out by these kinases are indicated by arrows and sites of phosphorylation are indicated as stars. These include phosphorylation of the ITAMs and CD19 (by LYN), BCAP and SLP-65 (by SYK), and PLCγ2 (by BTK). PI3K, depicted in blue, phosphorylates PIP_2_ to generate PIP_3_ at the membrane. PIP_3_ is indicated as a red circle and PIP_2_ by a black circle. PLCγ2 hydrolyzes PIP_2_ at the membrane to produce the secondary messengers IP_3_ and DAG which will induce a calcium flux into the cell and activate PKC, respectively. Further downstream signaling events that lead to an upregulation of target genes for B cell maturation and proliferation are not shown in this figure for simplicity.

Bruton’s tyrosine kinase (BTK) is a non-receptor tyrosine kinase that belongs to the TEC family. The five members of the TEC family kinases (BTK, ITK, TEC, TXK, and BMX) are expressed in various hematopoietic cell lineages and relay signals downstream of multiple immunological receptors. BTK has been most well studied in B cells in the context of B-cell receptor (BCR) signaling, but it also plays a role in macrophages, mast cells, and dendritic cells downstream of Fc receptors and Toll-like receptors (TLRs) (Weber et al., 2017; Pal Singh et al., 2018). Summarized in Figure 1, antigen binding to the BCR triggers the activation of a trio of tyrosine kinases: LYN, SYK, and BTK (Geahlen, 2009; Stepanek et al., 2013). LYN phosphorylates the immunoreceptor tyrosine-based activation motifs (ITAMs) within the Ig-α/β chains associated with the BCR as well as CD19. The phosphorylation of the ITAMs recruits SYK where it is activated. Activated SYK phosphorylates the B-cell adaptor protein for PI3K (BCAP) and the phosphorylation of BCAP and CD19 by these kinases promote activation of PI3K (Okada et al., 2000). Activated SYK phosphorylates SLP-65 (or BLNK) and activated PI3K phosphorylates PIP_2_ in the membrane to produce PIP_3_, which recruits BTK and its substrate phospholipase Cγ2 (PLCγ2) to the BCR complex. BTK association with PIP_3_ and SLP-65 activates BTK, which phosphorylates and activates PLCγ2 (Li et al., 1997). Activation of PLCγ2 in turn generates the second messengers, inositol 1,4,5-trisphosphate (IP_3_) and diacylglycerol (DAG), to induce a calcium flux and activate protein kinase C (PKC) leading into the MAP kinase pathway (Kurosaki, 2011). These signaling events ultimately lead to the upregulation of specific genes essential for B-cell maturation and proliferation.

BTK plays an instrumental role in B-cell development as mutations in the *BTK* gene have been linked to the primary immunodeficiency X-linked agammaglobulinemia (XLA) (Tsukada et al., 1993). XLA patients experience an increased susceptibility to bacterial infections due to an arrest in B-cell development at the pre-B cell stage. Enhanced BTK activity on the other hand is linked to the production of autoantibodies (Kersseboom et al., 2010; Kil et al., 2012; Corneth et al., 2017; Heukels et al., 2019) and this autoimmune phenotype is dependent on the catalytic activity of BTK (Kil et al., 2012). However, the nature of the autoantibodies produced: whether they are natural antibodies derived from B1 cells or immune antibodies is currently unclear (Satterthwaite, 2018). Taken together, it is clear that BTK function must be finely tuned to generate an appropriate immunological response. For this reason, BTK has been studied as a valid target for therapeutic development to tune the BCR signaling cascade. Specifically, BTK is a tissue specific target for inhibition in various B-cell lymphomas including diffuse large B-cell lymphoma (DLBCL) and chronic lymphocytic leukemia (CLL) (Davis et al., 2010; Herishanu et al., 2011); proliferation of these lymphomas strictly depends on the activation of the BTK signaling cascade. Inhibitors targeting BTK for treatment of these lymphomas have shown anti-tumor activity in lymphoma models, and three BTK specific inhibitors (Ibrutinib, Acalabrutinib, and Zanubrutinib) have FDA approval and are being used as a treatment option for patients. The specific role of BTK in B cell lymphomas has been reviewed elsewhere (Pal Singh et al., 2018).

BTK also plays an important role in innate immune signaling pathways in other hematopoietic cell lineages. BTK has been shown to function in antimicrobial responses downstream of TLRs and is involved in Fc receptor signaling (Weber et al., 2017). Given BTK’s role in innate immune signaling pathways, inhibitors of BTK are being investigated in treatment of rheumatoid arthritis (RA) (Di Paolo et al., 2011), and the 2019 novel coronavirus disease (COVID-19) caused by SARS-CoV-2 infection. Administration of Acalabrutinib, a second-generation inhibitor of BTK, improved oxygenation levels in over 70% of patients in a small patient cohort (Roschewski et al., 2020). In other reports, the effect of blocking BTK in the context of thromboinflammation in COVID-19 is considered (Nicolson et al., 2020; Siess et al., 2020). Moreover, as the SARS-CoV-2 virus spreads throughout the population, the number of patients already being treated with a BTK inhibitor that contract COVID-19 has increased. This has resulted in a number of recent reports detailing the effect of BTK inhibition during the course of COVID-19 infection (Chong et al., 2020; Lin et al., 2020; Thibaud et al., 2020; Treon et al., 2020). Currently, two clinical trials are underway to evaluate the efficacy of BTK inhibitors during COVID-19 treatment (NCT numbers: NCT04382586, NCT04346199, ClinicalTrials.gov).

## Inhibitors Targeting BTK

Due to the involvement of BTK in multiple immunological signaling pathways, there have been a host of inhibitors developed with different binding modes all targeting the kinase domain of BTK as a treatment option for B cell lymphomas and other BTK-reliant diseases. Supplementary Table 1 summarizes current BTK inhibitors classified by one of four binding modes: (1) covalent, irreversible, (2) covalent, reversible, (3) non-covalent, reversible, or (4) proteolysis targeting chimeras (PROTACs). Supplementary Figure 1 provides the chemical structures for those inhibitors for which this information has been disclosed. The following sections of this review will focus on inhibitors that have FDA approval, are at Phase 3 clinical trials, or represent a unique approach to targeting BTK. We aim to summarize select clinical data, describe the molecular mechanisms at work in Ibrutinib resistance mutations, and highlight the impact that specific inhibitors have on the conformational ensemble of full-length BTK.

## Ibrutinib and Resistance Mutations in BTK

**FIGURE 2:**
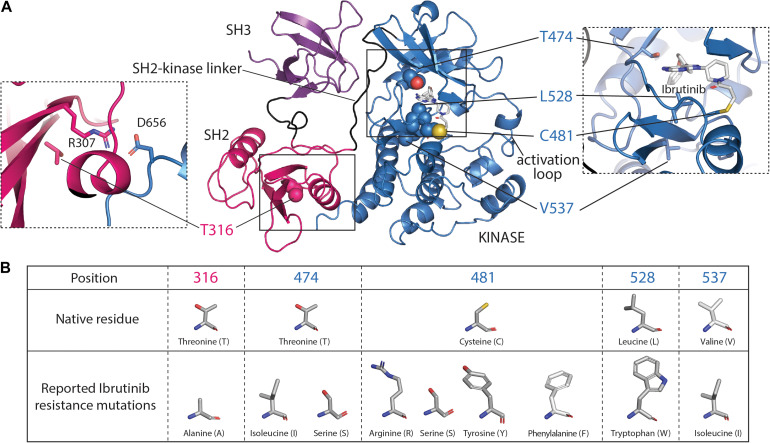
Ibrutinib resistance mutations mapped onto the SH3–SH2-kinase fragment of BTK. **(A)** The monomeric model of the SH3–SH2-kinase module of BTK is shown (Wang et al., 2015) with the SH3, SH2, and kinase domains colored purple, pink, and blue, respectively. The BTK kinase domain structure bound to Ibrutinib [Protein Data Bank (PDB) code: 5P9J] was used to place Ibrutinib in the SH3–SH2-kinase model. Ibrutinib resistance mutations that have been described at T316, T474, C481, L528, and V537 are shown. The left dashed box highlights the R307/D656 salt bridge in proximity to T316. The right dashed box highlights the Ibrutinib resistance mutations surrounding the active site. The boxed image here was taken from the Ibrutinib-bound kinase domain structure (PDB:5P9J). **(B)** The table presents structures of native amino acid residues and the corresponding Ibrutinib resistance mutations reported to date.

Ibrutinib (brand name Imbruvica) is the first-in-class covalent irreversible BTK inhibitor that was rationally designed to modify C481 after identifying a chemical scaffold that inhibited BTK kinase activity (Pan et al., 2007; Figure 2A). After demonstrating that Ibrutinib blocks B-cell activation downstream of the BCR in animal models of B-cell malignancy, Ibrutinib moved to randomized human clinical trials (Honigberg et al., 2010). Since its success in clinical trials, Ibrutinib has been approved for use in treatment of CLL, mantle cell lymphoma (MCL), Waldenström’s macroglobulinemia (WM), marginal zone lymphoma (MZL), and chronic graft versus host disease (cGVHD) and is in various stages of clinical trials for the treatment of other immune disorders. At the molecular level, Ibrutinib attaches to C481 within the kinase active site and acts as an ATP competitive inhibitor with an IC_50_ of 0.5 nM in a cell-free kinase assay (Honigberg et al., 2010). Ibrutinib is also known to bind to and inhibit multiple kinases including ITK, a TEC family kinase important in T-cell signaling (Dubovsky et al., 2013). The off-target binding of Ibrutinib is thought to contribute to undesired side effects namely bleeding events which is linked to platelet dysfunction upon Ibrutinib treatment (Shatzel et al., 2017).

While Ibrutinib has shown success in the clinic, there is an increasing occurrence of acquired resistance that is driven by either point mutations in the BTK protein itself or gain-of-function mutations in the BTK substrate PLCγ2 (Zhou et al., 2012). A 3-year cohort study found that acquired *BTK* mutations contributed to CLL progression while Ibrutinib was administered (Quinquenel et al., 2019). The most common Ibrutinib resistance mutation occurs at the site of covalent attachment, BTK C481. The C481S resistance mutation was first identified in the samples of five out of six relapsed CLL patients (Woyach et al., 2014) and has surfaced in other B-cell lymphomas such as WM and MCL supporting the notion that this mutation is a primary acquired resistance mutation that is a consequence of treatment with Ibrutinib (Chiron et al., 2014; Xu et al., 2017). The BTK C481S mutation does not affect the activity of BTK (Joseph et al., 2020) but mutation of this residue renders BTK insensitive to Ibrutinib due to the loss of the covalent bond to Ibrutinib (Cheng et al., 2015; Figure 2). The reversible binding of Ibrutinib by the BTK C481S mutant together with the rapid clearance of Ibrutinib from the plasma (mean half-life of 2–3 h, Advani et al., 2013; Davids and Brown, 2014), would significantly reduce the occupancy of BTK by Ibrutinib, leaving BTK uninhibited and signaling competent. In fact, a recent study showed that BTK occupancy is a critical factor that needs to be considered with the use of BTK covalent inhibitors (Sun et al., 2020; Thompson, 2020). A half dose of inhibitor taken twice daily by patients was found to have higher BTK occupancy and more effective inhibition than a single daily full dose of inhibitor. Sustained presence of a BTK inhibitor in plasma either by increasing inhibitor dosage frequency or by the use of inhibitors with a prolonged plasma half-life will be critical to counter resistance by the BTK C481S mutation.

Other acquired Ibrutinib resistance mutations surrounding the ATP binding site have been uncovered in relapsed patients (Figures 2A,B), including T474I/S, L528W, and V537I (Maddocks et al., 2015; Kanagal-Shamanna et al., 2019). While these kinase domain resistance mutations are thought to destabilize productive Ibrutinib binding, a distinct acquired resistance mutation, T316A, in the regulatory Src homology 2 (SH2) domain of BTK has been described (Sharma et al., 2016; Kadri et al., 2017; Figures 2A,B). The T316A Ibrutinib resistance mutation is unique in that it is the first and only described resistance mutation found outside the kinase domain of BTK (Figure 2A). The T316A mutation does not interfere with Ibrutinib binding but nevertheless confers resistance by permitting downstream phosphorylation signaling events (Sharma et al., 2016). Understanding how these point mutations contribute to Ibrutinib resistance at the molecular level is important as this information can aid inhibitor design to provide treatments for patients who acquire resistance. Recent solution nuclear magnetic resonance (NMR), hydrogen/deuterium exchange mass spectrometry (HDX-MS), and biochemical studies have revealed that the T316A mutation disrupts the autoinhibitory conformation of BTK (described in detail below) thereby increasing the active population of BTK, evading Ibrutinib inhibition (Joseph et al., 2020). There are similar reports of active site inhibitor resistance mutations disrupting autoinhibitory contacts in ABL, another multi-domain kinase (Saleh et al., 2017; Xie et al., 2020) suggesting that this mode of resistance may be a shared mechanism to bypass inhibition.

## Second Generation BTK Covalent Irreversible Inhibitors

Second generation covalent irreversible inhibitors have since been developed to increase specificity. There are several covalent irreversible BTK inhibitor candidates that have been developed including the FDA approved Acalabrutinib (brand name Calquence) (Barf et al., 2017) and Zanubrutinib (brand name Brukinsa) (Weaver and Jimeno, 2020), along with Spebrutinib (CC-292) (Evans et al., 2013), Tirabrutinib (Walter et al., 2016), Evobrutinib (Caldwell et al., 2019), and Tolebrutinib (Francesco et al., 2017). All mediate covalent attachment to BTK via C481, the Ibrutinib binding residue. At the biochemical level, kinetic experiments that compared a panel of covalent inhibitors (Ibrutinib, Spebrutinib, Acalabrutinib, and Tirabrutinib) revealed that while Ibrutinib had the most potent IC_50_ against BTK, it had the lowest selectivity for BTK when tested against a panel of kinases that possess the homologous cysteine binding residue in the ATP binding site (Liclican et al., 2020). The remainder of this section will focus on the second-generation irreversible covalent inhibitors that have either gained FDA approval or are currently in Phase 3 clinical trials.

Acalabrutinib was found to have an improved selectivity profile over other covalent inhibitors, Ibrutinib, and Spebrutinib (Barf et al., 2017). Computational modeling of Acalabrutinib in the ATP binding site of the BTK kinase domain predicts that Acalabrutinib makes more hydrogen bonds with the kinase domain compared to Ibrutinib (Barf et al., 2017). Because of its improved specificity and promising preclinical characterization in an animal model (Harrington et al., 2016), Acalabrutinib moved to clinical trials targeting various B cell lymphomas (Byrd et al., 2015; Wang et al., 2018; Girard et al., 2019). Acalabrutinib is currently FDA approved to use as a treatment for MCL, CLL, and small lymphocytic leukemia (SLL) patients. There is also a preclinical evaluation testing a combination of Acalabrutinib and a PI3K inhibitor in CLL mouse models to target two signaling proteins in the BCR signaling cascade (Niemann et al., 2017). A more extensive review of the preclinical and clinical data for Acalabrutinib has been published (Wu et al., 2016).

Zanubrutinib was rationally designed to improve both specificity for BTK and pharmacokinetic and pharmacodynamic properties such as oral absorption and target occupancy relative to Ibrutinib (Guo et al., 2019). Indeed, Zanubrutinib shows an improved selectivity against common off-targets of Ibrutinib: ITK, TEC, and epidermal growth factor receptor (EGFR) (Guo et al., 2019). Zanubrutinib swiftly moved into clinical trials for a variety of B cell lymphomas (Tam et al., 2019, 2020; Syed, 2020; Weaver and Jimeno, 2020) and has since been FDA approved as a treatment option for MCL patients. Compared to Ibrutinib’s binding mode, Zanubrutinib makes an extra hydrogen bond with M477 (Guo et al., 2019) which could explain its improved selectivity for BTK over other kinases (Kaptein et al., 2018).

Evobrutinib (M2951) and Tolebrutinib (SAR442168, PRN2246) are covalent irreversible BTK inhibitors currently active in Phase 3 clinical trials. Evobrutinib was rationally designed using B43, a moderately potent kinase inhibitor, as a starting structure to pursue structure activity relationship (SAR) drug design and has been evaluated in a Phase 2 clinical trial for multiple sclerosis (MS) (Caldwell et al., 2019; Montalban et al., 2019). This compound has since moved on to a Phase 3 clinical trial targeting the same disease (NCT04338022, ClinicalTrials.gov). At the molecular level, this inhibitor makes two sets of contacts within the kinase active site; a covalent irreversible bond to the sidechain of C481 and a non-covalent interaction with the selectivity pocket surrounding T474, the gatekeeper residue of BTK. The contact Evobrutinib makes in the T474 pocket is thought to impart greater specificity over other BTK inhibitors as a threonine at the gatekeeper position is found in only 20% of human kinases (Lou et al., 2012). Bulky, hydrophobic residues are more often located at this position in other kinases, so the smaller and more polar threonine side chain in BTK creates an additional pocket for ATP binding site inhibitors. Tolebrutinib (SAR442168/PRN2246) is unique from the other covalent irreversible inhibitors mentioned in that it has been designed to cross the blood brain barrier for BTK-dependent disease mechanisms relevant in the nervous system (Francesco et al., 2017). For this reason, Tolebrutinib is currently active in Phase 3 clinical trials for MS (NCT04411641 and NCT04458051, ClinicalTrials.gov).

## Covalent, Reversible BTK Inhibitors With Emphasis on Rilzabrutinib (PRN-1008)

While Ibrutinib and other covalent, irreversible inhibitors have shown success in the clinic, strategies to modulate residence time with irreversible kinase inhibitors is lacking. To overcome this challenge, design of covalent, reversible kinase inhibitors targeting non-catalytic cysteine residues in the protein has emerged. These covalent, reversible inhibitors usually contain a cyano-acrylamide scaffold that permits elimination of the cysteine residue upon unfolding of the tertiary structure (Serafimova et al., 2012). A covalent, reversible inhibitor targeting C481 in BTK, Rilzabrutinib (PRN1008), has been developed (Hill et al., 2015). In preclinical evaluations, Rilzabrutinib has shown a prolonged BTK occupancy time of over 100 h and also shows great specificity in targeting BTK over other common Ibrutinib off-targets (Hill et al., 2015). Early phase clinical trials indicated that Rilzabrutinib is well-tolerated in healthy volunteers (Smith et al., 2017) and showed a significant response in immune thrombocytopenia patients (Kuter et al., 2020). Rilzabrutinib has since moved to a Phase 3 clinical trial to evaluate its use in treating Immune Thrombocytopenia (NCT04562766, ClinicalTrials.gov). Other covalent, reversible BTK inhibitors are in preclinical evaluations and early stage clinical trials (Herter et al., 2018; Schnute et al., 2019).

## Non-Covalent, Reversible BTK Inhibitors With Emphasis on Fenebrutinib (GDC-0853)

Even though Ibrutinib and other covalent BTK inhibitors have been FDA approved for administration to patients, the occurrence of resistance mutations at the cysteine binding residue creates incentive to target BTK with non-covalent inhibitors. A study comparing a panel of covalent and non-covalent BTK inhibitors revealed that the non-covalent inhibitors tested were both more specific toward BTK and were able to target and inhibit Ibrutinib-resistant mutants of BTK (Johnson et al., 2016). There are various non-covalent inhibitors targeting BTK in different clinical stages of development, but our focus will be on the non-covalent inhibitor in Phase 3 clinical trials, Fenebrutinib (GDC-0853). The status of other non-covalent inhibitors is summarized in Supplementary Table 1.

GDC-0853 was rationally designed as a non-covalent BTK inhibitor with greater specificity than existing covalent inhibitors (Crawford et al., 2018). Preclinical characterization of GDC-0853 revealed that BCR signaling events downstream of BTK are inhibited and GDC-0853 has reduced off target effects; no inhibition of ITK or EGFR was observed (Reiff et al., 2018). Furthermore, GDC-0853 has also shown anti-tumor activity in patients harboring the C481S Ibrutinib resistance mutation (Byrd et al., 2019). These findings demonstrate that GDC-0853 could be a treatment option for patients who have relapsed after Ibrutinib and who possess resistance mutations at the covalent cysteine residue. A Phase 1 clinical trial is currently underway to study the safety of GDC-0853 in patients with relapsed CLL or other B-cell lymphomas (NCT01991184, ClinicalTrials.gov). Moreover, GDC-0853 is also being tested as a treatment option for RA, SLE, urticaria (Phase 2 trials), and MS (Phase 3 trial) (NCT02983227, NCT02908100, NCT03137069, NCT04544449, ClinicalTrials.gov).

## Alternative BTK Targeting Approaches

While small molecule inhibitors developed for BTK have shown success in the clinic, other strategies are being explored to combat resistance. An appealing strategy toward BTK inhibition is targeted degradation using PROTACs. PROTACs are bivalent ligands that are designed to specifically target a protein of interest for degradation via ubiquitination (Sakamoto et al., 2001). Recently, potent PROTACs (MT-802 and DD-03-171) have been developed for BTK. MT-802 is based on the Ibrutinib scaffold, while DD-030-171 is based on the CGI1746 scaffold (Buhimschi et al., 2018; Dobrovolsky et al., 2019). Both chimeric molecules trigger degradation of both wild-type and the C481S BTK in B cell lymphoma cells. However, the potency of PROTACs targeting BTK is context dependent where different cell types and tissues show different levels of BTK degradation even though exposure to the PROTAC is similar across these areas (Zorba et al., 2018). Nevertheless, targeted degradation of BTK could emerge as another tool in the arsenal for treating various immunological diseases. There are emerging PROTACs, including NX-2127 and NX-5948 that target BTK for degradation and show promise in preclinical evaluations (Robbins et al., 2020).

## BTK Autoinhibition

**FIGURE 3:**
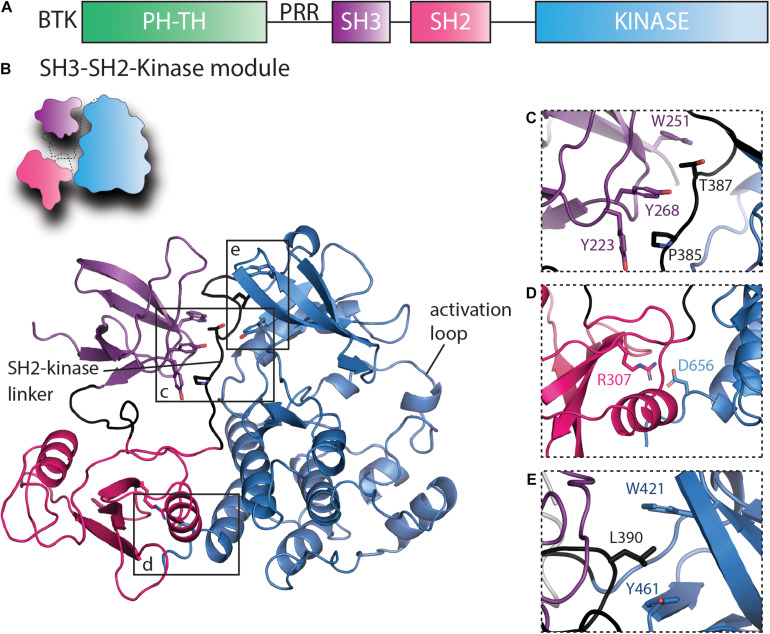
Regulatory interactions within the SH3–SH2-kinase module of BTK that maintain the inactive conformation. **(A)** Domain schematic of full-length BTK with the PHTH, SH3, SH2, and kinase domains colored green, purple, pink, and blue, respectively. The proline-rich region (PRR) between the PHTH and SH3 domains is labeled. **(B)** A cartoon of the autoinhibited BTK SH3–SH2-kinase model and the BTK SH3–SH2-kinase structure (Wang et al., 2015) are shown using the same domain colors as in panel **(A)**. The SH2-kinase linker and the activation loop are labeled, and boxes show the regions expanded in panels **(C–E)**. **(C)** The intramolecular interaction between SH3 domain residues Y223, W251, and Y268 and the SH2-kinase linker residues P385 and T387 stabilize the SH3 domain onto the distal side of the kinase domain (opposite the activation loop face). **(D)** The salt bridge contact between SH2 R307 and D656 in the kinase domain stabilizes the SH2 domain on the distal side of the kinase domain C-lobe and mimics the SH2/C-terminal tail phosphotyrosine interaction of the SRC kinases (Liu et al., 1993). **(E)** L390 in the SH2-kinase linker and W421 and Y461 in the kinase domain N-lobe form the hydrophobic stack.

All of the BTK inhibitors described above target the BTK active site situated between the N- and C-lobes of the kinase domain. BTK is a multidomain kinase composed of regulatory regions outside of the carboxy-terminal kinase domain. From the amino- to carboxy terminus is a Pleckstrin homology (PH) domain, a Tec homology (TH) domain, a proline-rich region (PRR), a Src homology 3 (SH3) domain, a SH2 domain, and finally the carboxy terminal kinase domain (Figure 3A). Unlike other families of non-receptor tyrosine kinases, BTK and the other TEC kinases have to date resisted crystallization in their full-length form. Nevertheless, fragment crystal structures and a range of biochemical and biophysical studies are revealing the molecular details of BTK regulation and set the stage to better understand how current BTK inhibitors affect full-length BTK to pave the way to develop allosteric approaches to modulate BTK function in disease.

Crystallographic studies performed by the Harrison and Kuriyan groups revealed autoinhibitory contacts within the SRC module (Shah et al., 2018) or SH3–SH2-kinase fragment of BTK (Wang et al., 2015). The structure of the SH3–SH2-kinase fragment of BTK shows a compact, SRC-like conformation with the SH3 and SH2 domains docked onto the back side of the kinase domain opposite the activation loop (Figure 3B). The conserved binding groove of the SH3 domain contacts the SH2-kinase linker (Figure 3C) while SH2 domain is docked onto the C-lobe of the kinase domain despite the absence in the TEC family of the phosphorylated tail found in SRC kinases (Figure 3D). Subsequent work applying solution NMR and HDX-MS to full-length BTK further probed these intramolecular interfaces and demonstrated that these interfaces maintain the autoinhibited, inactive conformation of BTK in solution (Joseph et al., 2017). This work also defined a specific salt bridge contact between R307 in the SH2 domain and D656 in the kinase domain (Figure 3D) that serves a role similar to the phosphorylated tail of the SRC kinases by stabilizing the autoinhibitory pose of the SH2 domain.

## SH3 and SH2 Domains Influence the Kinase Domain Regulatory Apparatus

The regulatory features within the kinase domain have been extensively reviewed elsewhere (Taylor and Kornev, 2011). Key conserved features include the αC helix, which transitions between the αC-out (inactive) and αC-in (active) conformations. A conserved Lys/Glu salt bridge stabilizes the αC-in or active conformation and transition to the αC-out or inactive state is accompanied by loss of the Lys/Glu salt bridge (Taylor et al., 2015). The activation loop also transitions between distinct conformers depending on the activation (and phosphorylation) status of the kinase domain. Phosphorylation of BTK Y551 in the activation loop triggers a conformational shift to favor opening of the activation loop, whereas the inactive state features a compact activation loop folded in toward the active site. This inactive activation loop conformation protects Y551 from phosphorylation and it is interesting to note that loop dynamics differ significantly between BTK and the T cell specific TEC family kinase ITK. The ITK activation loop strongly favors the inactive conformation compared to the BTK activation loop that readily samples the open and Y551 accessible state (Joseph et al., 2013). These findings suggest that the T cell kinase may be under greater regulatory control than BTK in B cells perhaps as a mechanism to limit spurious T cell activation. Additional regulatory features within the kinase domain include the DFG motif, the regulatory spine, and the hydrophobic stack. Briefly, when the DFG motif adopts a DFG-in conformation, the phenylalanine of this motif participates in assembling the regulatory spine which is essential for TEC family kinase activation (Joseph et al., 2010).

The hydrophobic stack is a set of three residues [in BTK W421 and Y461 from the kinase domain N-lobe and L390 from the SH2-kinase linker (Figure 3E)] that when assembled is thought to stabilize the autoinhibited form of the kinase (Von Raußendorf et al., 2017). Thus, contacts between the SH3 domain and the SH2-kinase linker serve to not only protect the SH3 binding groove from engaging with exogenous ligands but also position the side chain of L390 to complete the hydrophobic stack on the back on the kinase domain N-lobe. In the TEC and SRC family kinases, it has been demonstrated that disruption of this hydrophobic stack results in exchange of ADP for ATP promoting full activation (Von Raußendorf et al., 2017).

The domain arrangement in the BTK autoinhibited structure also shields the phosphotyrosine binding pocket of the SH2 domain from interactions with other ligands (Figure 3D). The salt bridge between SH2 and kinase domains ties up R307 preventing that side chain from engaging phosphotyrosine ligands. However, compared to the intramolecular phosphotyrosine/SH2 interaction present in autoinhibited structures of SRC family kinases (Liu et al., 1993), this region of autoinhibited BTK is likely more dynamic and prone to release from the kinase domain. Indeed, crystal structures of both isolated TEC family SH2 domains (Joseph et al., 2012) and the crystal structure of the BTK SH3–SH2-kinase fragment (Wang et al., 2015) show the SH2 domain adopting a domain swapped dimer structure. Whether this mode of dimerization is physiologically relevant is not known. Small angle X-ray scattering (SAXS) data suggested quite early that BTK adopts an extended arrangement of its domains (Márquez et al., 2003), however, the functional state that this extended conformational state reflects is unclear. More recent work has demonstrated that a BTK SH2 specific binding protein abrogates the kinase activity of BTK by blocking a predicted SH2/kinase domain interface required for activation (Duarte et al., 2020; Jeong et al., 2020). The interface between SH2 and kinase domains in TEC family and other tyrosine kinases is known to play a critical role in enhancing kinase activity beyond the isolated kinase domain (Nagar et al., 2003; Joseph et al., 2007; Filippakopoulos et al., 2008; Lamontanara et al., 2014). However, the precise structural features of the interface between the SH2 and kinase domains within active BTK are not yet determined but could eventually provide a route toward targeting BTK to disfavor activating contacts for specific, allosteric inhibition of BTK.

## A Defining Feature of BTK and the TEC Family Kinases, the PHTH Domain

**FIGURE 4:**
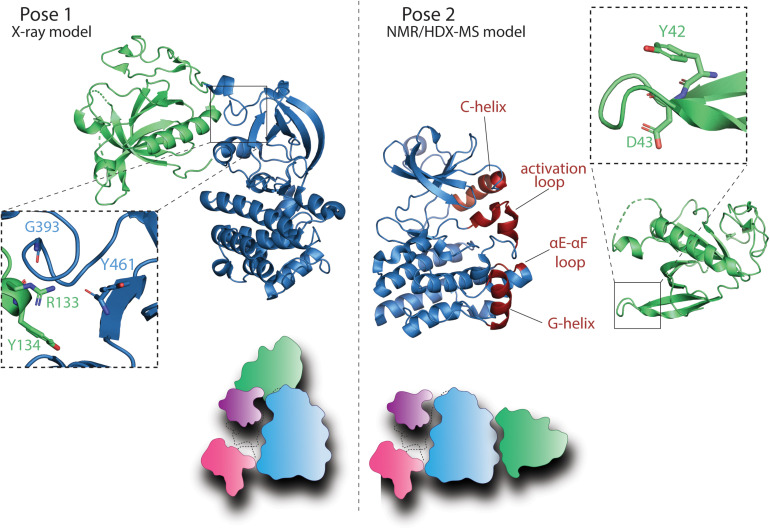
BTK PHTH autoinhibitory interactions. Pose 1: Model derived from the crystal structure of a tethered BTK PHTH-kinase construct (PDB: 4Y93) reveals contacts between the PHTH domain and the kinase domain N-lobe [partially overlapping with the autoinhibitory site of the SH3 domain (Wang et al., 2015)]. Domain colors are as in Figure 3A. The expanded region (dashed box) shows the interaction between R133 and Y134 in the PHTH domain and G393 and Y461 in the kinase domain. The cartoon depiction shows the PHTH/kinase interaction in the context of the autoinhibited SH3–SH2-kinase structure described in Figure 3. Pose 2: Solution NMR, HDX-MS, and biochemical data reveal another autoinhibitory interaction between the PHTH and the activation loop face of the kinase domain (Devkota et al., 2017; Joseph et al., 2017; Amatya et al., 2019). The PHTH domain residues involved in this autoinhibitory pose are Y42 and D43 (dashed box) while the structural elements in the kinase domain reported to mediate this interaction include the αC-helix, the activation loop (A-loop), the αE–αF loop, and the αG-helix (colored in red). The cartoon depiction shows this PHTH/kinase interaction in the context of the autoinhibitory SH3–SH2-kinase structure described in Figure 3.

Two distinct autoinhibitory contacts are described in the literature for the BTK PHTH domain. X-ray crystal structures of a tethered BTK PHTH-Kinase construct revealed a docking site for the PHTH domain on the N-lobe of the BTK kinase domain (Figure 4, Pose 1) while solution NMR approaches revealed a different PHTH domain docking site on the C-lobe of the kinase domain (Figure 4, Pose 2). Pose 1 (Figure 4) involves specific contacts between R133 and Y134 in the PHTH domain and the N-lobe of the kinase domain. Importantly, this crystallographically determined structure shows the PHTH domain adopting the “Saraste dimer” (Hyvönen and Saraste, 1997), a structure that has been associated with binding of the BTK PHTH domain to the plasma membrane following production of PIP_3_ from PIP_2_ by PI3K (Chung et al., 2019). Molecular dynamics simulations suggest that the BTK PHTH Saraste dimer is stabilized at the membrane by binding multiple PIP_3_ lipids and mutations that are known to have disease relevance destabilized the dimer interface (Wang et al., 2019). Furthermore, elegant studies using supported lipid bilayers provide evidence that the peripheral site originally identified in the crystal structure of the BTK PH domain bound to IP_6_ (Wang et al., 2015) stabilizes membrane association (Chung et al., 2019). This requirement for the occupancy of both PIP_3_ sites for activation of BTK suggests that the PH domain is sensitive to the concentration of PIP_3_ in the membrane. In a resting cell, due to the broad conformational ensemble of BTK, the PH domain could be sampling the membrane for PIP_3_ and activation is only triggered when the PIP_3_ concentration surpasses a certain threshold and both canonical and peripheral sites are occupied, stabilizing dimerization of BTK at the membrane.

The BTK PHTH domain in Pose 2 (Figure 4) blocks access of substrate to the kinase domain active site (Amatya et al., 2019) and the residues of the PHTH domain that mediate this autoinhibitory interaction (Y42 and D43) (Devkota et al., 2017; Joseph et al., 2017) are the same residues that mediate the Saraste dimer interface suggesting that this autoinhibitory pose is likely mutually exclusive with the active BTK dimer. The PHTH domain of ITK also blocks the kinase domain active site, impeding access to the activation loop (Devkota et al., 2017). Moreover, phosphatidylinositol binding to the PHTH domain inhibits the interaction between PHTH and kinase domains in solution (Devkota et al., 2017). Interestingly, a similar PH/kinase domain interface has been described for AKT although in this case a small molecule inhibitor seems to promote the domain–domain interaction (Barnett et al., 2005; Calleja et al., 2009; Wu et al., 2010). More recent structural work combining segmental isotopic labeling and NMR spectroscopy has revealed the complexity of the AKT regulatory system (Chu et al., 2018, 2020) and given the domain differences between BTK and AKT it is not yet clear how similar the autoinhibitory role of the shared PH domain will be. Nevertheless, the PH domain of AKT is subject to inhibition by small molecules (Meuillet, 2011; Parikh et al., 2012; Gao et al., 2018; Kang et al., 2018; Yang et al., 2019; Zhang et al., 2020) and therefore provides a template for a similar approach to targeting BTK. Beyond simply targeting the lipid binding function of the BTK PHTH domain (Yoon, 2014), the more mature picture of BTK regulatory interfaces now permits new approaches to target the BTK PHTH/kinase interfaces to allosterically control BTK function.

## The BTK Conformational Ensemble

**FIGURE 5:**
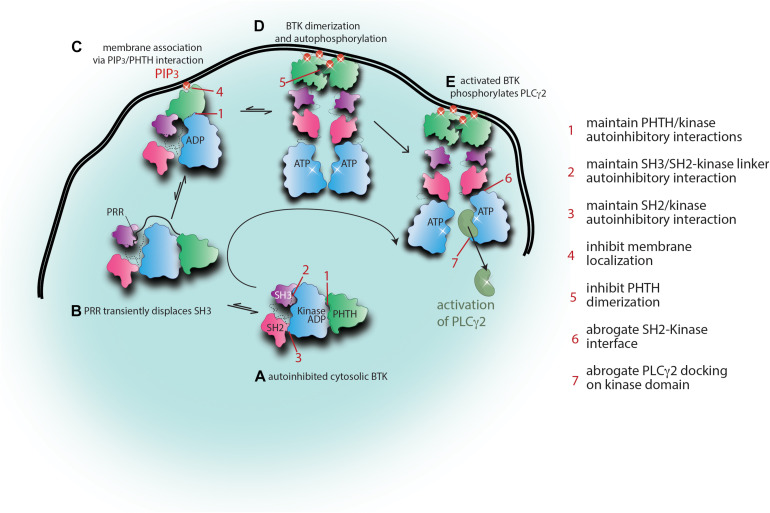
Alternate approaches to targeting BTK. Snapshots of the activation scheme for BTK provide several areas to target for allosteric therapeutic development. Current knowledge of the BTK activation scheme is summarized here with cartoon representations. The domain colors are as in Figures 3, 4. **(A)** Briefly, autoinhibited cytosolic BTK adopts a compact domain arrangement with the PHTH docked onto the kinase domain blocking the active site (Pose 2) and the SH3 and SH2 domains adopting a SRC-like conformation. **(B)** Displacement of the SH3 domain from the distal side of the kinase domain by the proline-rich region (PRR) promotes transient opening of the autoinhibited structure which would promote regulatory domain accessibility to exogenous ligands (including PHTH domain sampling of the PIP_3_ content in the plasma membrane). **(C)** Initial binding of the PHTH domain to PIP_3_ might lead to domain rearrangement consistent with the Pose 1 PHTH/kinase interaction. **(D)** Once PIP_3_ levels in the membrane surpass the required threshold, BTK engages PIP_3_ via the canonical and peripheral binding sites in PHTH and BTK dimerization at the membrane is stabilized. Rearrangement of the regulatory domains ensues, the SH2 domain transitions to its activating position on the N-lobe, the hydrophobic stack is disassembled triggering exchange of ADP for ATP in the kinase domain allowing autophosphorylation. **(E)** Activated BTK phosphorylates its substrate PLCγ2. Seven areas for potential allosteric therapeutic development are listed. These areas encompass stabilizing autoinhibited BTK (points 1, 2, and 3) and/or disfavoring activating or substrate contacts (points 4, 5, 6, and 7).

It is likely that conformational plasticity is the reason the TEC family kinases have to date resisted crystallization in their full-length form. Indeed, solution NMR data for full-length BTK shows multiple resonances for BTK W395 consistent with the protein adopting multiple distinct conformations that are in slow exchange on the NMR timescale (Joseph et al., 2017). Ultimately, more work is needed to generate a full picture of the BTK activation trajectory, but we can consider the “ensemble” of different conformational states that have been characterized to date. A compact, fully autoinhibited model of BTK can be described based on the crystal structure of the SH3–SH2-kinase fragment and the solution work placing the PHTH domain in Pose 2 across the activation loop of the kinase domain (Figure 5A). In this state, each of the regulatory domains are prevented from binding to other target ligands, the hydrophobic stack is fully assembled favoring ADP bound to the active site, and the activation loop is protected from phosphorylation. NMR and biochemical data (Andreotti et al., 1997; Laederach et al., 2002; Joseph et al., 2017) have previously invoked a role for the PRR in binding the SH3 domain and transiently displacing the SH3 domain and likely the SH2 domain from the fully autoinhibited state (Figure 5B). This transient opening of the BTK autoinhibited structure might favor phospholipid association once PIP_3_ is present at the membrane (Figure 5C). Once membrane associated, BTK will dimerize via the Saraste dimer and membrane association will be further stabilized by PIP_3_ binding to both the canonical and peripheral sites on the PH domain (Figure 5D; Chung et al., 2019). Upon membrane dimerization it is likely that further rearrangement of the SH2 domain to create contacts with the kinase domain N-lobe will stabilize the kinase domain in its active state (Figure 5D). Once activated by phosphorylation of Y551 on the activation loop, BTK is poised to phosphorylate its substrate PLCγ2 (Figure 5E). For the related TEC kinase, ITK, a substrate docking site on the C-lobe of the kinase domain has been described (Xie et al., 2013) and it is possible that the BTK/PLCγ2 enzyme/substrate pair shares a similar mechanism to achieve substrate specificity.

The emerging model for BTK conformational transitions and interactions in an activated B cell provides a number of possible targets to develop allosteric approaches to BTK inhibition. As already discussed, the drug resistance that is associated with active site inhibitors such as Ibrutinib may be solved by combinations of Ibrutinib and small molecules that act at separate locations on the BTK protein. In particular, resistance mutations remote from the active site such as BTK T316A might be especially vulnerable to combination therapies that include an allosteric inhibitor that compensates for the mutation induced shift toward the active state. Taking a broader view of potential allosteric inhibitors, Figure 5 suggests seven distinct sites that might prove “druggable” in future work. The most compact autoinhibited conformation of BTK may be stabilized by small molecules that target the PHTH/kinase interface (1), the SH3/SH2-kinase linker contact (2), and/or the SH2/kinase interaction that in the native autoinhibited structure is weakly held by a single salt bridge (3). Targeting the membrane bound state of BTK, small molecules that prevent PHTH/PIP_3_ engagement (4) or PHTH dimerization (5) might prove useful in truncating BTK signaling. Interfering with the activating interaction between the BTK SH2 and kinase domains (6) and/or preventing the PLCγ2 substrate from docking onto the BTK kinase domain (7) might provide yet more avenues toward inhibiting BTK function. As additional structural details and mechanistic insights into BTK regulation continue to emerge the list of potential allosteric target sites will increase.

## Direct Inhibition in the BTK Active Site Modulates the Full-Length BTK Conformational Ensemble

Due to challenges associated with co-crystallization of drug bound multidomain kinases, the majority of structural work on kinase inhibitor binding tends to be limited to the local effects of the inhibitor on the regulatory features within the kinase domain itself (αC-helix, DFG motif, and activation loop). For example, one study of BTK active site inhibitors found that different small molecule inhibitors differentially sequester the activation loop Y551 and inhibitors that do not sequester this residue exhibit reduced inhibition against Fcε receptor signaling compared to BCR signaling (Bender et al., 2017). Understanding precisely how a given inhibitor affects different BTK mediated signaling pathways is extremely important in a clinical setting and this level of understanding must extend beyond the kinase domain. An increasing volume of work is being published on understanding the molecular level influence of small molecule inhibitors on full-length kinases (Skora et al., 2013; Tong et al., 2017; Chakraborty et al., 2019; Fang et al., 2020). Detailed evaluation of a panel of BTK inhibitors has demonstrated that different active site inhibitors exert a range of distinct dynamic and conformational effects on the remote non-catalytic regulatory domains (Joseph et al., 2020). Ibrutinib proves the most interesting case as covalent binding of Ibrutinib to the BTK active site promotes release of both the SH3 and SH2 domains, as well as the SH2-kinase linker, from their autoinhibitory poses. This is in contrast to the other active site inhibitors; neither CC-292 (also covalent), GDC-0853, nor CGI1764 (both non-covalent) significantly alter the conformational ensemble of the full-length BTK autoinhibitory conformation. The disruption of the BTK autoinhibitory conformation upon Ibrutinib binding makes the BTK regulatory domains available for interaction with upstream and downstream ligands which could promote BTK kinase independent function and/or have dominant negative effects. In fact, in the context of treating B cell lymphomas, PLCγ2 ibrutinib resistant variants are hyper-sensitive to activation regardless of BTK’s kinase activity suggesting that the kinase-independent functions of BTK might be responsible (Wist et al., 2020). Blocking BTK kinase activity alone therefore may not be sufficient in the successful treatment of disease states. Future development of BTK inhibitors and the selection of BTK inhibitor used to treat disease states will need to carefully consider the impact the inhibitor has on the conformation of the full-length protein.

## Combination Targeting of Orthosteric and Allosteric Sites

This review has focused on current approaches to BTK inhibition in the clinic, the state-of-the-art knowledge surrounding BTK regulation at the molecular level, and how the FDA approved BTK inhibitor Ibrutinib induces long range structural effects on BTK that might affect drug efficacy in certain contexts. Targeting BTK at allosteric sites could provide better specificity but perhaps more importantly might counteract the conformational influence of resistance mutations or even binding of Ibrutinib and second-generation inhibitors related in structure to Ibrutinib. Targeting both an orthosteric and allosteric site within the same kinase has been achieved for other systems (Eide et al., 2019; To et al., 2019). Furthermore, an explicit conformational connection between an allosteric and active site of a kinase has been shown for phosphoinositide-dependent kinase-1 (PDK1). In that work, HDX-MS data show that binding of ATP destabilized the allosteric PIFtide pocket, making that site more amenable to PIFtide binding (Ghode et al., 2020). In another kinase, cyclin-dependent kinase-2 (CDK2), there is demonstrated positive cooperativity in allosteric inhibitor binding when certain orthosteric inhibitors are present in the ATP site that enhance the allosteric inhibitor’s affinity (Faber et al., 2020). Following these examples, it is intriguing to consider whether the conformational consequences of Ibrutinib binding to BTK might create new allosteric target sites that can be exploited to ultimately gain complete control over BTK function in the catalytic and regulatory domains. As work progresses it will be important to consider how candidate allosteric inhibitors against BTK affect active site structure and dynamics.

## Conclusion

Multidomain kinases present many challenges to the full structural characterization needed to elucidate regulatory mechanisms and the effects of inhibitor binding. Multidisciplinary approaches spanning crystallography to solution methods including NMR and HDX-MS to cellular assays are pushing the field forward and as a result we are becoming better equipped to understand and combat disease inducing mutations and drug resistance. It will be exciting to witness the impact of fundamental biophysical characterization on kinase inhibitor development and the use of these inhibitors in a clinical setting.

## Author Contributions

LK wrote the manuscript and made the figures. AA and RJ edited the manuscript and contributed to some of the writing. All authors contributed to the article and approved the submitted version.

## Conflict of Interest

The authors declare that the research was conducted in the absence of any commercial or financial relationships that could be construed as apotential conflict of interest.

## Abbreviations

BCAP: B cell adaptor protein for PI3K
BTK: Bruton’s tyrosine kinase
CDK2: cyclin dependent kinase-2
cGVHD: chronic graft versus host disease
CLL: chronic lymphocytic leukemia
DAG: diacylglycerol
DLBCL: diffuse large B-cell lymphoma
EGFR: epidermal growth factor receptor
HDX-MS: hydrogen/deuterium exchange mass spectrometry
IP_3_: inositol 1,4,5-trisphosphate
MCL: mantle cell lymphoma
MS: multiple sclerosis
MZL: marginal zone lymphoma
NMR: nuclear magnetic resonance
PDB: Protein Data Bank
PDK1: phosphoinositide-dependent-kinase-1
PH: Pleckstrin homology
PHTH: Pleckstrin homology-Tec homology
PI3K: phosphoinositide 3-kinase
PIP_2_: phosphatidylinositol (4,5)-bisphosphate
PIP_3_: phosphatidylinositol (3,4,5)-trisphosphate
PKC: protein kinase C
PLC γ 2: phospholipase C γ 2
PROTAC: proteolysis targeting chimera
RA: rheumatoid arthritis
SAR: structure activity relationship
SH2: Src homology 2
SH3: Src homology 3
SLE: systemic lupus erythematosus
SLL: small lymphocytic leukemia
SLP-65: SH2 domain-containing leukocyte protein of 65 kDa
SYK: spleen tyrosine kinase
TH: Tec homology
WM: Waldenström’s macroglobulinemia.
