# Supplementary material for: Reining in BTK: Interdomain Interactions and Their Importance in the Regulatory Control of BTK
Source: Front Cell Dev Biol. 2021 Jun 23;9:655489. doi: 10.3389/fcell.2021.655489 (PMC8260988; doi:10.3389/fcell.2021.655489)
Supplement: Supplementary file 1 [file Data_Sheet_1.PDF]

Covalent, irreversible BTK inhibitors

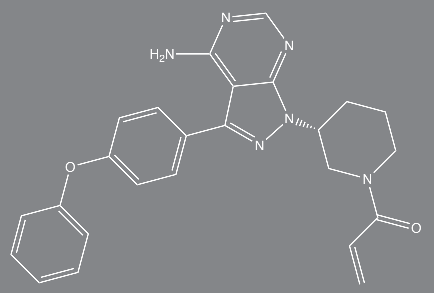

Ibrutinib

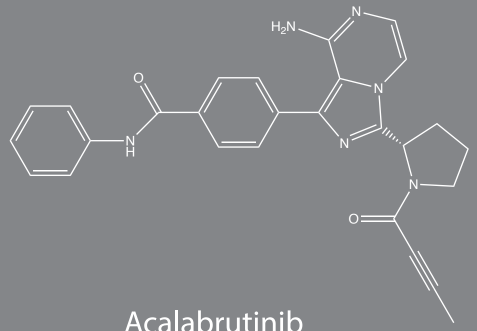

Acalabrutinib

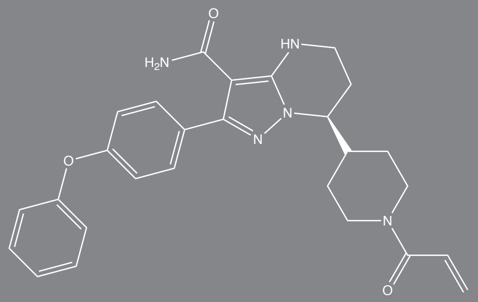

Zanubrutinib

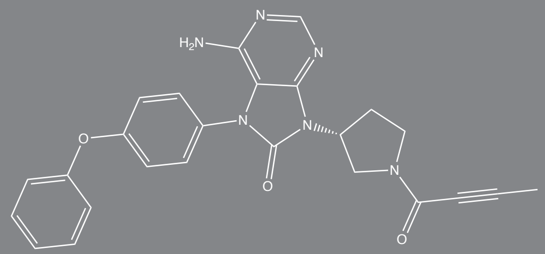

Tirabrutinib

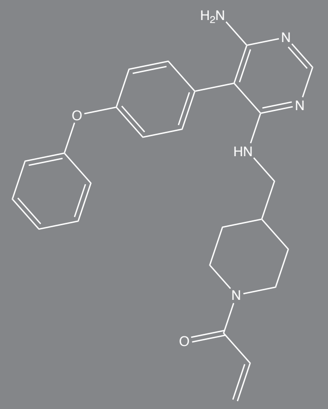

Evobrutinib

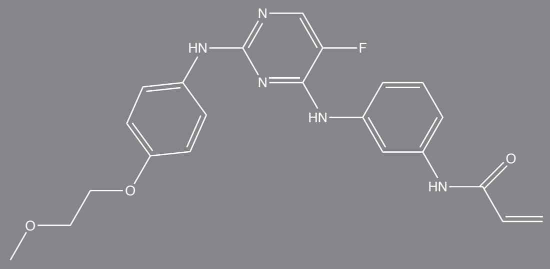

Spebrutinib

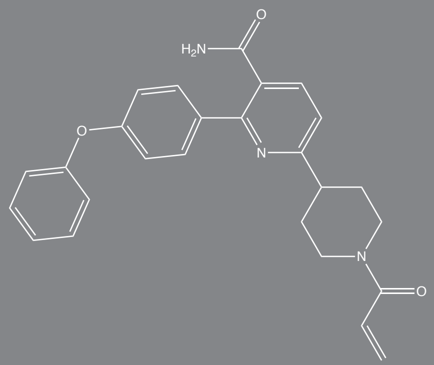

Orelabrutinib

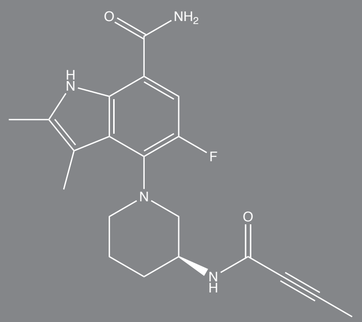

Branebrutinib

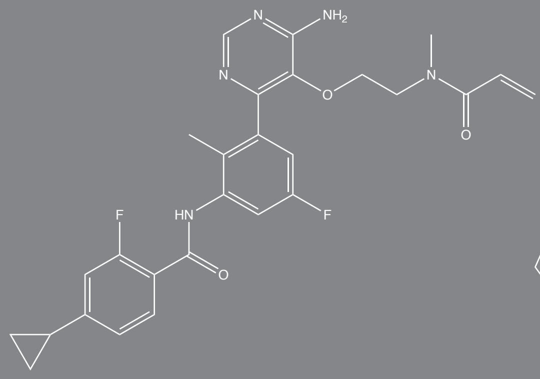

Remibrutinib

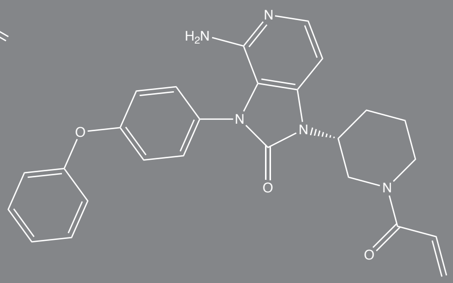

Tolebrutinib

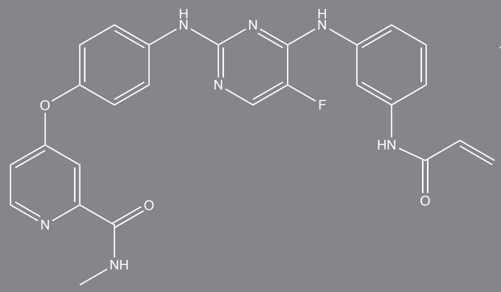

CNX-774

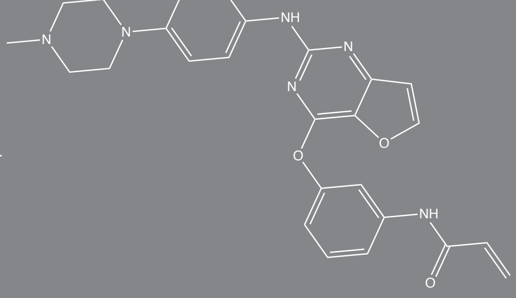

Poseltinib

Covalent, irreversible BTK inhibitors, cont'd.

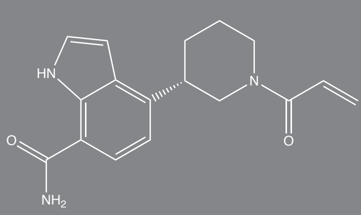

Elsubrutinib

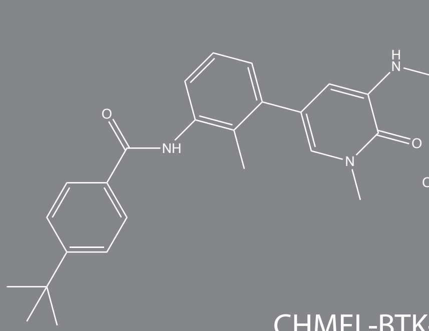

CHMFL-BTK-01

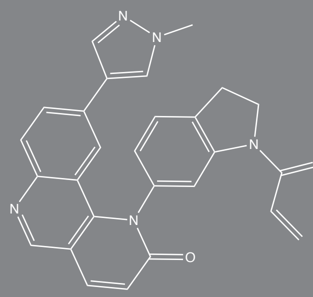

QL47

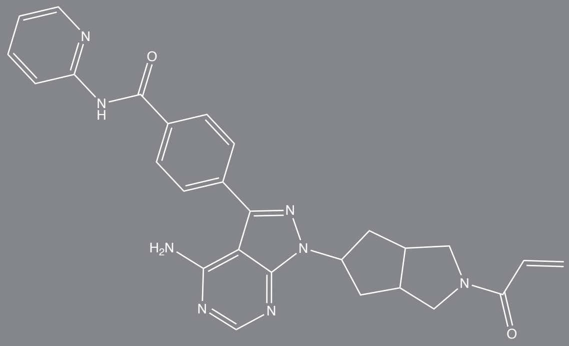

ZYBT1

Covalent, reversible BTK inhibitors

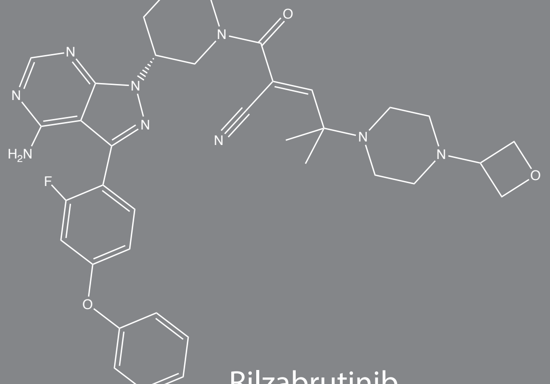

Rilzabrutinib

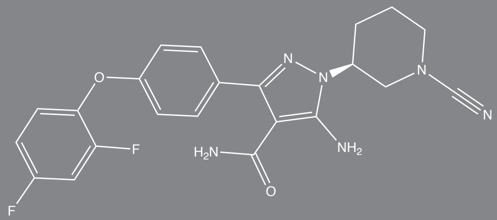

PF-06250112

Noncovalent, reversible BTK inhibitors

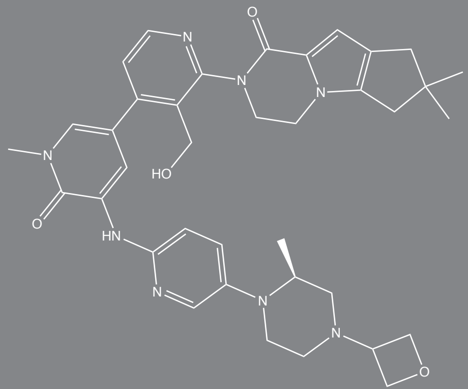

Fenebrutinib

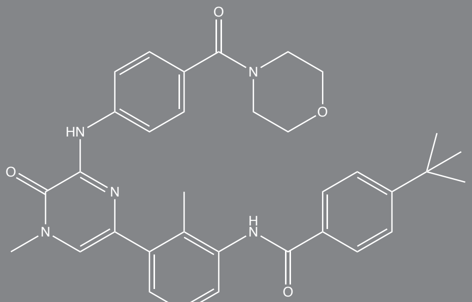

CGI1746

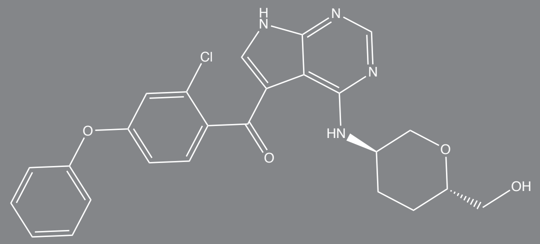

ARQ 531

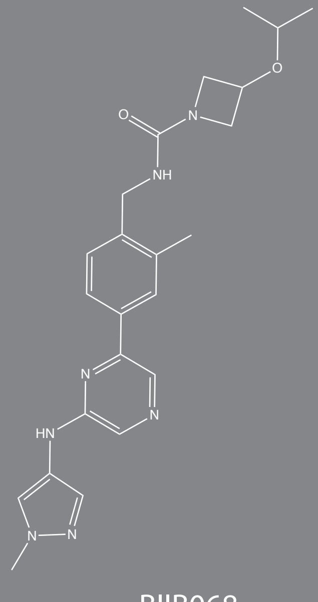

BIIB068

Noncovalent, reversible BTK inhibitors, cont'd

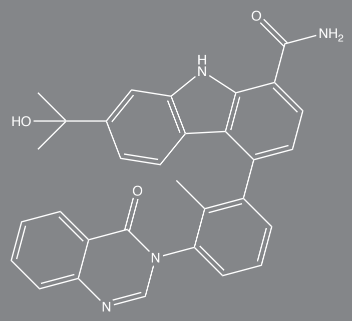

BMS-935177

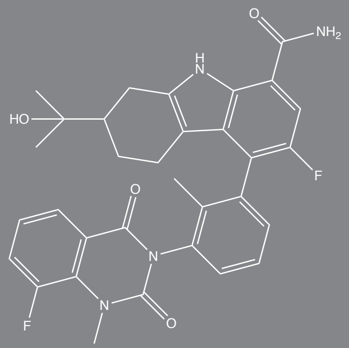

BMS-986142

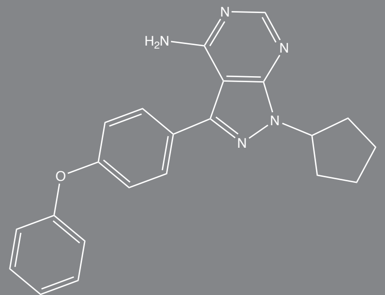

PCI-29732

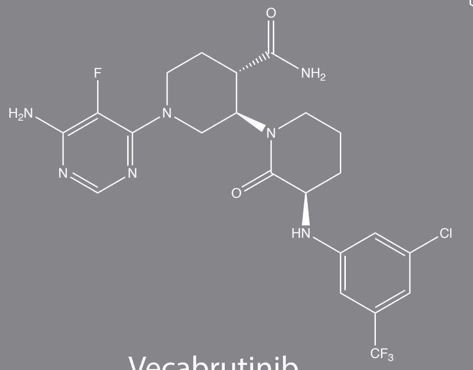

Vecabrutinib

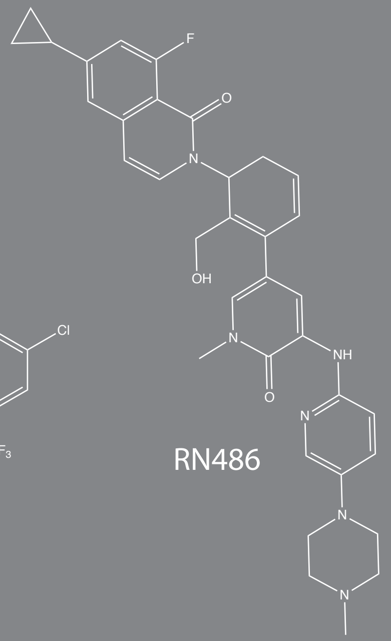

RN486

Proteolysis targeting chimeras (PROTACs) for BTK degradation

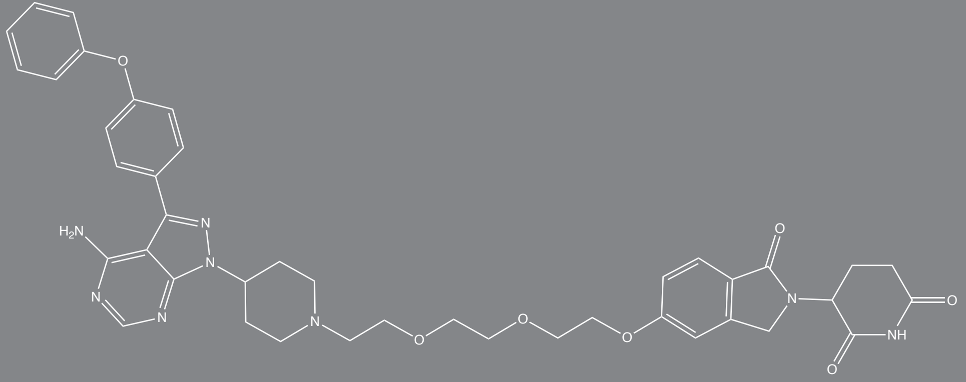

SJF620

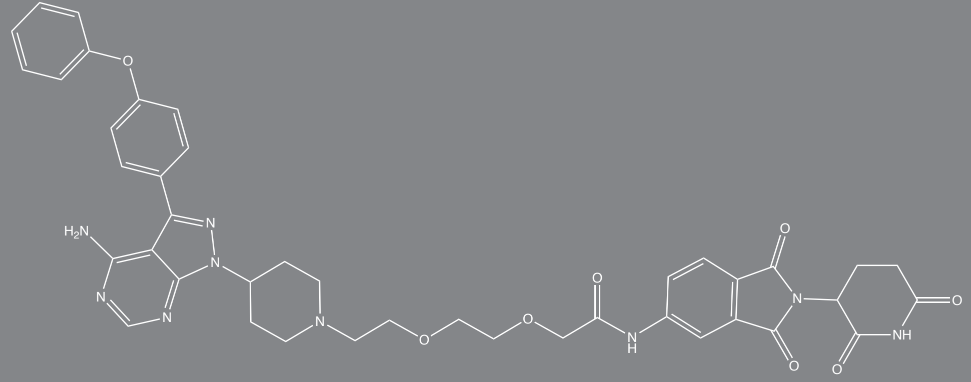

MT-802

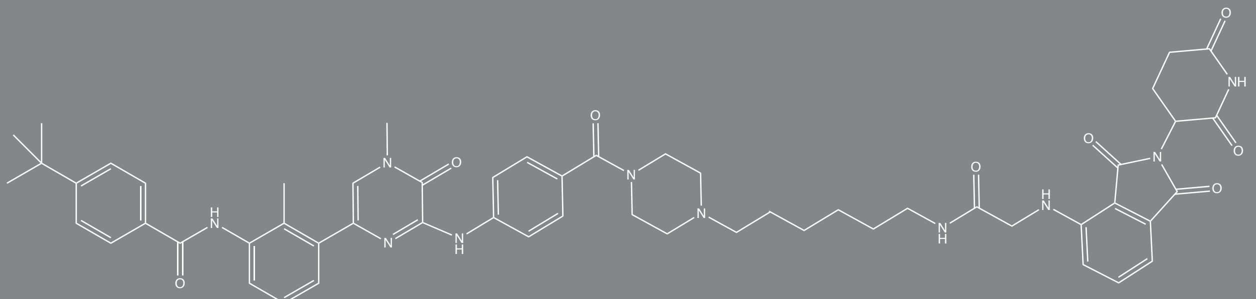

DD-03-171
